# Proposal of a Selection Protocol for Replication of Studies in Sports and Exercise Science

**DOI:** 10.1007/s40279-022-01749-1

**Published:** 2022-09-06

**Authors:** Jennifer Murphy, Cristian Mesquida, Aaron R. Caldwell, Brian D. Earp, Joe P. Warne

**Affiliations:** 1 Centre of Applied Science for Health, Technological University Dublin, Tallaght, Dublin Ireland; 2 Natick, MA USA; 3 Yale-Hastings Program in Ethics & Health Policy, Yale University and The Hastings Center, New Haven, CT USA; 4 Uehiro Centre for Practical Ethics, University of Oxford, Oxford, UK

## Abstract

**Introduction:**

To improve the rigor of science, experimental evidence for scientific claims ideally needs to be replicated repeatedly with comparable analyses and new data to increase the collective confidence in the veracity of those claims. Large replication projects in psychology and cancer biology have evaluated the replicability of their fields but no collaborative effort has been undertaken in sports and exercise science. We propose to undertake such an effort here. As this is the first large replication project in this field, there is no agreed-upon protocol for selecting studies to replicate. Criticism of previous selection protocols include claims they were non-randomised and non-representative. Any selection protocol in sports and exercise science must be representative to provide an accurate estimate of replicability of the field. Our aim is to produce a protocol for selecting studies to replicate for inclusion in a large replication project in sports and exercise science.

**Methods:**

The proposed selection protocol uses multiple inclusion and exclusion criteria for replication study selection, including: the year of publication and citation rankings, research disciplines, study types, the research question and key dependent variable, study methods and feasibility. Studies selected for replication will be stratified into pools based on instrumentation and expertise required, and will then be allocated to volunteer laboratories for replication. Replication outcomes will be assessed using a multiple inferential strategy and descriptive information will be reported regarding the final number of included and excluded studies, and original author responses to requests for raw data.

**Supplementary Information:**

The online version contains supplementary material available at 10.1007/s40279-022-01749-1.

## Key Points

**Table:** 

Replication increases or decreases confidence in claims by providing additional evidence for those claims.
Previous replication projects in psychology and cancer biology have contributed to concerns of a replication crisis in science.
The proposed protocol transparently describes the methods for selecting studies to replicate for the first large replication project in sports and exercise science.
Selection criteria considers the year of publication and citation rankings, research disciplines, study types, the research question and key dependent variable, study methods and feasibility.

## Introduction

An important goal of science is to advance knowledge, through the generation of novel empirical claims that are robustly supported [1, 2]. For an experimentally derived claim to be robustly supported, it should, in principle, be replicable. Ideally, repeatedly using comparable analyses after collecting new data, in adequately powered studies conducted across varied relevant contexts [3]. The original and replication study must be similar in all “theoretically relevant dimensions” such as study design, methods, and materials [4]. Replication is therefore defined as retesting a claim using new data and comparable analyses, while reproducibility uses the same data and analyses [5]. Consequently, one goal of replication is to bolster our collective confidence in the veracity of novel claims by providing diagnostic evidence about those claims [5, 6]. Although replication is most valuable when the existing understanding for a theory is weakest [5], it can also update boundaries of a claim to further develop theories [7, 8]. Researchers have focused attention on how replication studies should be conducted and how their results should be interpreted [9–12]. Yet, there has been little discussion about the factors influencing study selection for replication and even less of a consensus on what those factors should be [1]. Moreover, researchers are limited by energy and resource constraints; selecting studies for replication that have the most potential to teach us something useful is therefore paramount.

Large-scale replication projects have been undertaken by, among others, the Open Science Collaboration in the form of the Reproducibility Project: Psychology, the Many Labs Project, and the Reproducibility Project: Cancer Biology [13–15]. These projects contributed to the impression that there is a replication crisis in science due to the inability of the researchers to successfully replicate many selected effects. The Reproducibility Project: Psychology selected 100 effects to replicate from three leading psychology journals and reported a replication rate of 36% (when using statistical significance, *p* < 0.05, as the criterion for assessment). The Many Labs Project, by contrast, successfully replicated 10 of 13 effects. The Reproducibility Project: Cancer Biology reported that 92% of their replication effect size estimates were smaller than the original estimates. This percentage should be approximately 50% if the original effect sizes were accurately estimated [15]. Altogether, these findings suggest that there is large variation in the replicability of effects and considerable room for improvement in the respective scientific fields.

Given the interdisciplinary nature of sports and exercise science, and its overlap with the psychological sciences, there is reason to believe the sports and exercise science field faces similar replication issues [16]. The replicability of sports and exercise science research has yet to be examined despite the identification of concerns within the field [17–19]. The goal of the present project is to sample a range of topics across the field of sports and exercise science for an initial estimation of the replicability of those findings. This is the first natural step in the assessment of replicability of the field; therefore, the project will attempt to replicate numerous effects once rather than multiple replications of a specific effect or claim derived from theory. As this is the first large scale replication project in the field of sports and exercise science, there has been no previous discussion on study selection protocols in the literature.

Whilst the replication projects in the field of psychology are encouraging initiatives in science, some have criticised the Open Science Collaboration’s selection protocol for replication as it chose effects that were not representative of the field and which were not selected at random from within the identified set of relevant studies [20–22]. Similarly, the Many Labs selection protocol was criticised for selecting “easy” to run studies because they had to be short and suitable for online presentation. The Many Labs coordinators were also criticised for allowing each replication team to select their study from a pool of 13 studies (researcher selection bias) and using the last study in each article for replication, which can lead to problematic hypotheses selection [20, 21]. Due to these criticisms, there are calls for a more formalised selection process for replication in which the benefits of the replication outweigh the costs [23]. These criticisms and calls for a formalised selection process show the need to produce a randomised selection protocol in sports and exercise science in order to provide an initial overview of replicability in the field.

Throughout the discussion on the selection of replication studies in other fields, three factors recur: statistical, theoretical/practical and methodological aspects of the chosen studies [24, 25]. Studies might be chosen for replication based on a need to reduce existing uncertainty around the statistical soundness of reported findings, based, for example, on inconsistencies in the statistical results, inflated effect sizes with wide confidence intervals, conflicting previous results, and the prevalence of questionable research practices (e.g. p-hacking) [1, 23]. Studies could also be targeted for replication based on their theoretical or practical value, as indicated by their scholarly or public impact (e.g., Altmetric attention score or citation impact) [25]. Finally, studies might be chosen for replication due to methodological concerns suggesting a need for further scrutiny. These concerns include (but are not limited to) low statistical power and threats to internal validity from various biases [25, 26].

Guidelines were proposed to determine what studies are most worthwhile to replicate based on a Bayesian decision-making framework [24, 27–29] or the proposed replication value of the study (i.e., what we stand to learn by replicating the finding) [1, 23, 30]. Although Isager and colleagues admit no guidelines can “*provide a single set of rules for deciding what to replicate in all circumstances*” [1], there should be some justification for the selection of studies to replicate. For a large-scale replication project such as that proposed here, the selection of studies to replicate should be based on the aims of the project, namely, to provide an initial estimate of the overall replicability of studies within the entire field. There is a risk of selection bias in larger projects whereby studies are selected based on the belief they are easy to replicate or that the replication study results will differ from the original study results [9, 31]. To avoid such bias, once a set of studies is identified that is representative of the field, with resource availability and feasibility constraints factored in, the studies that are selected for replication should then be picked at random. This requires a more formalised process to ensure that those criteria are met. The authors of this paper intend to undertake a large-scale replication effort in sports and exercise science. Thus, as preliminary work towards this effort, we propose and transparently describe a selection protocol for replication studies.

## Methods

This protocol proposes the use of several steps in the selection of a pool of studies for replication, which are summarised in Table 1. All decisions and justifications at each step during this process are transparently reported.

**Table 1:** Overview of inclusion criteria for selecting studies to replicate

	Inclusion criteria
Year of publication	Quartile 1 journals according to www.scimagojr.com
Citation ranking	Original research articles published in 2015–2021
Research discipline	Applied sport and exercise training disciplines including:
	Applied sport and exercise psychology
	Applied sport and exercise nutrition
	Applied sport and exercise biomechanics
	Applied sport and exercise physiology
	Injury prevention
Study type	Experimental or quasi-experimental studies
	Independent variable is manipulated to determine the effect on a dependent variable
	Pairwise, independent or crossover study designs
	Statistically significant primary outcome
Research question and key variable	Clearly defined research aim or hypothesis
	Key variable is stated in the abstract, first or primary hypothesis or defined as important by the original author
Study methods: sample	Final sample reported
	Details of sample characteristics
	Alternatively, information available through original author contact
	First instance of author using the same sample in a research article
Study methods: equipment/software	Manufacturer details of equipment/software in original study
	Alternatively, information available through original author contact
Study methods: boundary conditions	Clear statements on boundary conditions
	Alternatively, information available through original author contact

### Year of Publication and Citation Rankings

The theoretical relevance of a topic or original study is important during the consideration of selecting studies to replicate [1, 24, 30]. When selecting studies to replicate, one should aim to investigate relevant research questions that are of current interest to the field [1, 9, 23–25, 30]. Therefore, recent research will be selected for replication, which we arbitrarily define as studies that were published in the last five years, from the date of each stage of the replication effort. A focus on recent research could also increase the probability of obtaining raw data from the original study authors when it is requested.

In a similar manner, journal quartile ranking as provided by www.scimagojr.com was also considered. An observational task was undertaken before the writing of this protocol on journal quartile rankings. We reviewed citation patterns in sports and exercise science literature and found 65% of researchers cited articles published in quartile 1 journals, even when they published in lower ranked journals (see online resource for full details). Consequently, we will only include studies that have been published in quartile 1 journals due to the frequency of their citation.

### Research Disciplines

The initial goal of this project, as previously discussed, is to provide an overview of the replicability of sports and exercise science research. As it is beyond the scope of this project to replicate all areas of sports and exercise science, the research area of *applied sports and exercise science* has been proposed for replication due its practical value. The following operational definitions are proposed: applied sports and exercise science is the study of the changes in human performance in response to physical activity, exercise and sport. The disciplines include applied sports and exercise nutrition, injury prevention, applied sports and exercise physiology, applied sports and exercise psychology and applied sports and exercise biomechanics. Applied sports and exercise physiology is the study of the adaptations to the neuromuscular, cardiovascular, endocrine and thermoregulatory system that affect sports and exercise performance. Applied sports and exercise psychology is the study of psychological factors affecting sports and exercise performance. Applied sports and exercise biomechanics is the study of human movement in sports and exercise. These disciplines have an expected utility due to perceived public interest [23] and are considered to be of practical value due to the uses of the topics or claims published by practitioners in the field [25]. Studies which include clinical populations and outpatients will be excluded from the study pool due to focus on applied sports and exercise science outcomes.

Journals in quartile 1 according to www.scimagojr.com (as of 30th May, 2022) that publish research in applied sports and exercise science include: American Journal of Sports Medicine, Sports Medicine, British Journal of Sports Medicine, Medicine and Science in Sports and Exercise, Journal of Physiology, International Journal of Sports Physiology and Performance, Journal of Science and Medicine in Sport, Scandinavian Journal of Medicine and Science in Sports, Psychology of Sport and Exercise, Journal of the International Society of Sports Nutrition (Open Access), International Journal of Sport Nutrition and Exercise Metabolism, Journal of Applied Physiology, Nutrition and Metabolism, Journal of Strength and Conditioning Research, Journal of Sport and Exercise Psychology, Journal of Sports Sciences, European Journal of Applied Physiology, and European Journal of Sport Science. The inclusion of these journals is based solely on the aims and scopes provided on the journal homepage where it includes any of the disciplines of applied sports and exercise science that have been described previously. Thereafter, the individual papers from within each journal will be further screened to only include papers that meet this applied sports and exercise science criteria.

### Study Types

The studies included for selection must be experimental or quasi-experimental quantitative studies, whereby an independent variable is manipulated to determine the effect on the dependent variable, in pairwise, independent or crossover study designs and across two or more groups. To compare results across studies, we will include studies that report a statistically significant outcome for the key variable (i.e., reject the null hypothesis based on a *p*-value falling below the stated alpha criterion in the original study; for criticisms, see [32]). Studies involving Bayesian analyses are excluded. Our focus on statistically significant key variables (e.g., counter-movement jump height) stems from the findings of Twomey and colleagues [33]. They reported an 80% positive results rate in sports and exercise science, i.e., most researchers in the field are reporting statistically significant findings. Consequently, we focus on testing a selection of these claims in our replication project. In order to be selected, studies must also have reported the mean and confidence intervals, an effect size or provide the raw data to calculate this information. This criterion excludes reviews of any type (e.g., systematic, narrative, and educational), meta-analysis, consensus statements, opinion pieces or commentaries, editorials, case studies, conference proceedings, study protocols, perspectives, and methodological reports. As the effect size from the original study will be compared with the replication effect size to assess the replication outcome, original studies must include sufficient information to calculate measures of effect size, or the raw data must be supplied by the original authors. If the effect size cannot be calculated due to insufficient information and non-response from original authors for raw data, the studies will be excluded. The assessment of replication outcomes is discussed in further detail later.

### Research Question and Key Variable

The original study should have a clearly defined research question or primary hypothesis for replication, which is stated in the introduction. If there are multiple hypotheses or dependent variables, the first or primary hypothesis with the theoretically most central statistically significant effect will be considered. Otherwise, the key variable should be stated in the abstract, aims, or defined as important by the author’s language [24]. Alternatively, if the key variable is the only variable applicable to applied sport science, it will be chosen for purposes of attempted replication. If the authors do not state a hypothesis and there are multiple dependent variables that could be chosen, the key variable of the study will be randomly selected by the project leads using www.random.org. To be randomly selected, the key variable must be stated in the abstract or aims.

### Study Methods

This project aims to conduct close replications, whereby methods for the replication study are based closely on the original study, with any differences being those that are unavoidable, e.g., a different sample [9]. Therefore, the original study should ideally have clear methods and high-quality reporting in order to be replicated. The replication study methods must be meticulously planned to be as close as possible to the original methods and made available for critique from the authors of the original study to eliminate random error and alternative explanations for replication outcomes [21]. It is most likely that original authors will need to be contacted for additional information to achieve a close replication. If there are many methodological issues that cannot be addressed without a large deviation from the original study methods, the study cannot be considered for replication as it would not constitute a close replication [9]. Similarly, the study will be excluded if major confounding variables or flaws are identified, and a close replication cannot be undertaken, as these types of studies have little value. Therefore, the replication team will not try to make improvements to the original study procedure [21]. The replicability of the original methods and the communication process with the original authors will be fully documented in the final project paper.

#### Sample

Ideally, the original study should include full details of the sampling methods, e.g., convenience sample from university student pool, and full sample of characteristics, e.g., age, height, body mass, training history, etc. Otherwise, sample details should be available from original authors. Studies will not be included if they do not state a final sample size number or include missing sample characteristics, and in cases where this information cannot be obtained from original authors, because a close replication would be unachievable. If it appears the study sample was measured on more than one occasion for different publications (i.e., separate publications of the same dataset), and by the same corresponding or first author, only one of the studies will be included in the replication pool. The sample descriptive statistics will be reviewed for this detail. The replication studies will have sample sizes larger than the original study as we aim to conduct high-powered replication studies (see Sect. 2.6). The details provided by the original study on sampling method and sample characteristics will inform the recruitment for the replication study. It is essential to control for potential confounding variables in the sample that could affect the outcome of the replication study [34].

#### Equipment/Software

All equipment and software used in the original study should ideally be listed with manufacturer details and full details of the versions used. Alternatively, the original authors can provide these and other key details required. In cases where this information is unavailable and original authors have not responded, the project leader will attempt to match the equipment with replication laboratories based on the information available for close replication. If a close replication cannot be achieved, the study will be excluded. Details of laboratory equipment and software availability will be acquired during the volunteer registration process. The allocation of studies to laboratories is fully described in a later section. The differences in equipment and software between the original and replication study will be fully documented as per “The Replication Recipe” [9].

It can be argued that an effect tested using unreliable and invalidated equipment or software will lead to a high variance in results affecting the internal and external validity of the study, thereby, the replication outcome [20]. However, this is an essential element of assessing the replicability of the sports and exercise science field. Replicating only those studies that have reported all reliability and validity statistics of the equipment used, would hugely narrow the pool of eligible studies, because the reporting of this information is inconsistent at best in sports and exercise science literature. Accordingly, the reporting of reliability and validity statistics will not be used as an inclusion criterion in this protocol.

#### Boundary Conditions

Identification of boundary conditions for the replication study will ensure that variables that were considered irrelevant a priori in the original study, but had an effect on the outcome post hoc, are controlled [9]. In some situations where an additional variable is necessary to make the replication “close”, it will be measured, but only the selected key variable will be used to assess the replication outcome. However, in practice, these additional variables or confounding factors may not be easily identifiable. The decision to measure an additional variable will be made by the replication team where it is obvious. Otherwise, it should be decided in communication with the original study authors. This process will be transparently reported in the supplementary materials of the final replication paper.

As it is recommended to make the replication study methods available to the original authors, they will be contacted to maximise replication quality and to provide any missing details that were present in the original study methods. The following outcomes are anticipated [35]:

1. Authors respond and give details to endorse the adequacy of replication methods.
2. Authors have concerns about the replication methods that the replication team has tried to address; if unresolved, the concerns should be listed a priori in the preregistration document.
3. Authors do not respond.

In case of a non-response, the replication team should make an informed decision to proceed with the replication methods unless there are concerns that the deviation will cause ambiguity and affect the replication outcome. Each replication study will be submitted as a preregistration before data collection commences, and be available publicly online at https://osf.io/3vufg/.

### Statistical Power

Traditionally, a priori statistical power calculations are based on original effect size estimates. However, these estimates are typically inflated due to publication bias and low study power, and they may possibly be the result of questionable research practices, e.g., *p*-hacking and multiple analyses [36, 37]. Similarly, the sample effect size is presumed to be a point estimate; however, there is a distribution for this value as it is not known with certainty [36]. Thus, a sample effect size estimate could be an overestimation of the true population effect size. Consequently, statistical power based on this effect size estimate can be lower than intended [38]. Therefore, a power analysis will be conducted a priori to each replication study using the “BUCSS” R package, which adjusts for publication bias and effect size uncertainty [39] in the statistical software R (version 1.4.1106) [40]. A limitation of this method is that *t*-values and *F*-values from the original study are required for the calculation. As a result, where *t*-values and *F*-values are not available, or cannot be calculated, the lower limit of the observed effect size confidence interval will be calculated from the original study. This method will be used to establish the sample size required for a power of ≥ 95% to detect the reported effect size of interest with the original study alpha criterion for a two-tailed test using the same study procedures. Otherwise, for reasons of feasibility, the observed effect size from the original study or doubling of the original sample size will be used to for sample size calculations if the other power analysis methods are not possible. The replication sample size will not be smaller than the original sample size. There is a possibility that doubling the original sample size will lead to underpowered replication studies. If an original study has a *p*-value of 0.03, the replication power is estimated to be 50% when using this method [41]. Although this could be highly informative for the statistical power of our field and potential shrinkage of effect size estimates [20].

### Feasibility

The allocation of replication studies to laboratories will consider feasibility in terms of the equipment, software, expertise required, and the sample to be recruited. Replication studies will not be allocated to original authors or laboratories and they will not assist other laboratories with data collection. Therefore, only laboratories that can feasibly run the replication study should be allocated that study. For example, some studies will be run comfortably within a laboratory’s consumables budget or there may be external funding available, whereas financial constraints will prevent another laboratory from conducting the replication study. Similarly, studies which will require specific equipment or software will be matched to those laboratories where the equipment is readily available in the laboratory or available to purchase through a consumables budget. If it is not possible to purchase the equipment for financial reasons, it shall be excluded. It is essential to match replication studies to laboratories in this protocol to avoid excluding studies with expensive equipment or specific expertise which would decrease the representativeness of the replication study pool. Otherwise, studies would be included because they are “easy” to run as was criticised in the Many Labs project [21]. These factors should be discussed on an individual basis between the project lead and replication teams.

Retrospective studies or those requiring long-term data collection of greater than 12 weeks will not be included in the study selection pool. Only acute or short-intervention studies (≤ 12 weeks) will be included.

Original studies that require complex statistical analysis or modelling will be excluded, as it is recommended that the key dependent variable can be evaluated with a single inference test (e.g., linear modelling or mixed modelling) [35]. Thus, studies that contain single inference tests, such as the *t-*test and the main effect of an *F-*test will be included. For studies that use factorial *F* tests, the key dependent variable will not be selected for replication if the interaction effect is significant; we will only select a key variable with a non-significant interaction and a significant main effect. Finally, studies that have clearly used the incorrect statistical test for their study design will not be included in the final study pool. This will be descriptively reported as discussed below.

### Final Selection

**Fig. 1:**
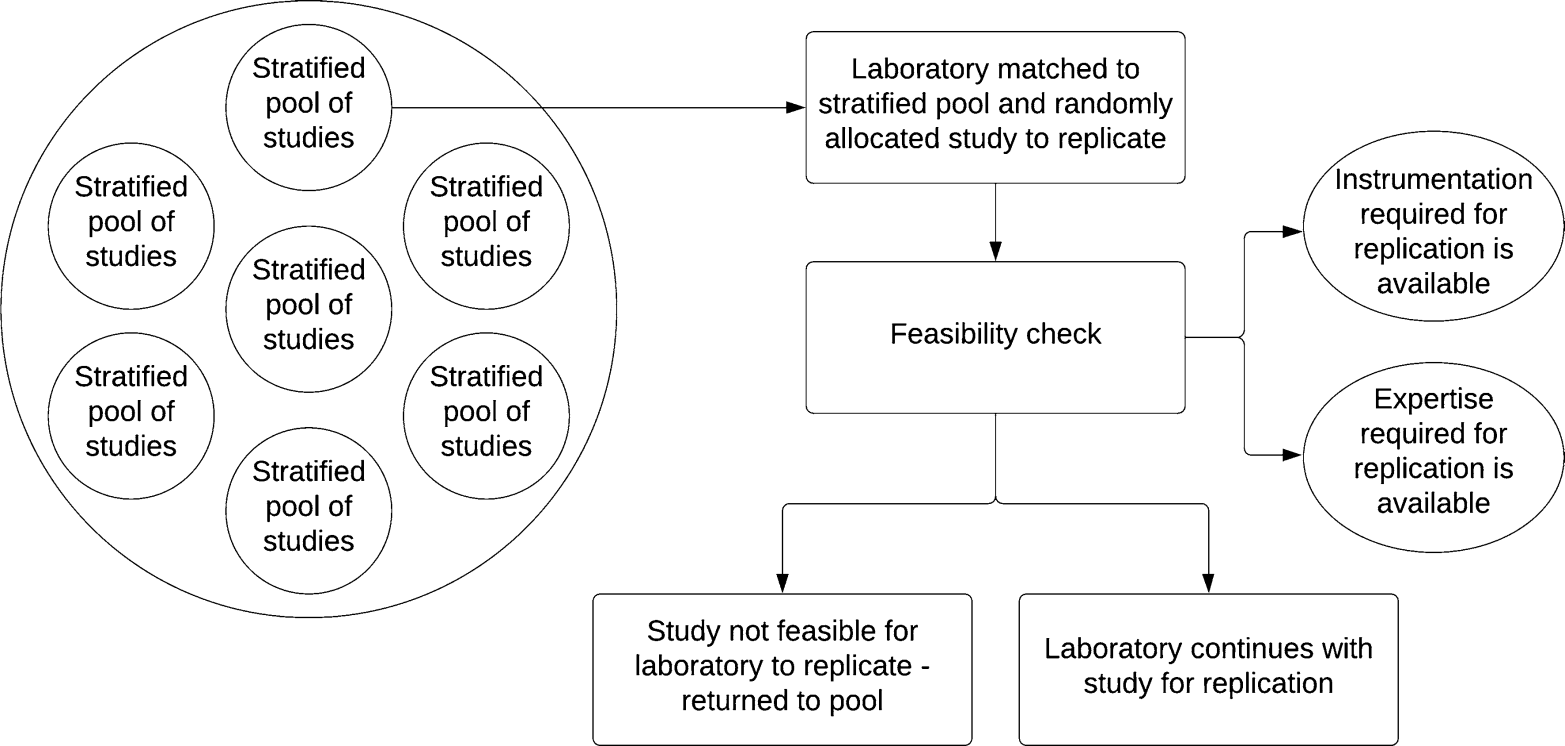
Allocation of replication studies to volunteer laboratories

Following the above steps for the inclusion or exclusion of studies, a study pool of feasible replications will be created. The allocation of studies to the laboratories will follow a stratified random sampling method. The availability of equipment, software, their versions and expertise in the volunteer laboratories will be identified early in this process. A pool of studies will then be created based on the equipment, software, and expertise required to run the replication study. From this stratified pool, a study will be randomly selected for allocation to a corresponding suitable laboratory for replication using a random number generator (https://www.random.org) (Fig. 1). This process of study selection will be recorded and linked to the study appropriately. The suitability of the laboratory will only be determined by available equipment, qualifications, and resources within, and not on laboratory preference. If the study drawn from the pool is not feasible for the selected laboratory to replicate (due to sample size recruitment issues, costs or availability of equipment and software, or lack of required expertise for measurements where required), the study will be returned to the pool and another study will be randomly selected. This process can be repeated until a feasible study for that particular laboratory is selected, although, this situation should be infrequent due to the stratification of studies. These are the only reasons for returning a study to the pool in order to reduce the potential of selection bias. This process will be reported in the supplementary materials of the final project paper.

## Data Analysis

A set criteria must be established for assessing replication outcomes, which will be provided in the main project report before commencing data collection. There are many different methods of evaluating replication outcomes and there is no singular method that is wholly advantageous over another. Rather, multiple inferential strategies should be used to overcome the limitations of each stand-alone criterion [42]. Therefore, we will use null hypothesis significance testing to determine if the replication effect size is statistically significant, and in the same direction as the original effect size. In addition to visually plotting the original and replication effect size estimates and their confidence intervals, we will quantitatively compare the effect size magnitudes using z-tests. This statistical test is useful as it can be uniformly applied to assess replication outcomes regardless of the original study analysis, i.e., *t-* or *F*-test. The overall project team leads, including their statistical collaborators, will conduct the data analyses.

Further descriptive information of the included and excluded studies according to our criteria will also be reported. This information will include the final number of studies included in each stratified pool, studies that were excluded at abstract and at full-text stage and the reason for exclusion based on the criteria provided, studies where the original authors must be contacted for raw data and methodological details, and original author response rates. The reporting of this information will be essential to future replication teams and for reviewer guidelines to determine what information is commonly omitted in sports and exercise science articles. This is an important opportunity to inform authors of the importance of data, code, and materials documentation in a way that would promote both replicability and reproducibility in the field.

## Discussion

Our study selection protocol will be used in a planned large-scale replication project in sports and exercise science. To echo Isager and colleagues (2020), it is not a definitive guideline or set of exhaustive criteria for the selection of studies to replicate, and is not without its limitations. There are facets of other study selection protocols that have not been implemented in this protocol. These include the assessment of replication value via citation count, methodological or statistical issues in the original study, and a Bayesian decision-making framework.

The decision of whether or not to replicate an original study can be influenced by the replication value or academic impact of a research topic or effect [1, 9, 23–25, 30, 43]. However, replication value was not directly quantified in this protocol for a number of reasons. Studies with a high citation impact (greater than 100 citations) could be replicated to prevent flawed or fraudulent findings from being unchallenged over time [43, 44]. Yet, this citation threshold is likely to be unrealistic for research published in the last five years. It is also biased towards the older research of the pool, and in addition may be biased towards trending topics as opposed to those with the most theoretical merit. Additionally, claims that have previously been successfully replicated or else (effectively) falsified [27] in systematic reviews or meta-analyses will have lower replication value. Nevertheless, it is difficult to specify how similar the research question must be in advance of selecting studies. Finally, a study can also have high replication value if there are calls to replicate it by authors in the field or by the original authors to replicate their own findings [25, 34]. However, previous calls for replication refer to field-wide replication rather than specific study or effects [19, 45]. As citation impact is not considered in this protocol, and due to issues quantifying replication value, we decided that replication value should not be used as a criterion for our protocol.

Methodological concerns within the original study can increase the need for replication due to doubt over the study conclusions. It can be argued that original studies with inflated effect sizes and wide confidence intervals, resulting from small sample sizes, should be replicated to increase the precision of the intervals and increase the certainty in the effect being studied [25]. However, methodological concerns about the original study were not included as a selection criterion for replication in our protocol for several reasons. The thresholds for what constitutes an inflated effect size or wide confidence intervals would need to be decided by the study selection team, which could inadvertently lead to bias. Replicating a study because it has an inflated effect size and large confidence intervals can also potentially evoke selection bias due to the doubt over the original effect [31]. The desire to narrow confidence intervals for an effect could be used as a selection criterion for replication when it is within a researcher’s personal interest and field, or for a singular replication study rather than a large-scale effort. Overall, due to the potential bias traps here, it was deemed that methodological concerns should not be included as a selection criterion in this protocol.

In the future, it will be important to replicate studies with inconsistent statistical results or those with the suspicion of questionable research practices [24]. Yet, the intent of this protocol is to remain as unbiased and representative as possible in selecting eligible studies [31]. Specifically selecting studies due to the suspicion of questionable research practices, or inconsistent statistical results only, could result in a final study pool that is less representative of applied sports and exercise science research. Similarly, the final outcome of this large replication project would be even less representative due to the focus on poorly designed studies. Accordingly, as the purpose of this project is to provide an unbiased initial assessment of replicability in sports and exercise science research, there is not a focus on statistical concerns due to the suspicion of questionable research practices in the original studies.

Our protocol uses original study *p*-values for statistical significance criterion when selecting studies to replicate. The *p*-values, however, in the context of null hypothesis significance testing, can be problematic because neither a statistically significant effect or non-significant effect is concrete evidence of a true effect or lack thereof [24]. Also, this method never allows us to gather evidence in favour of the null hypothesis; only evidence against it [46]. Along with the high positive result rate in the field [33], this protocol focuses on statistically significant effects for reasons of feasibility. An attempt to replicate a non-significant effect (where *p* > 0.05) would require infeasible sample sizes more than 16 times the original to obtain 80% replication power [41]. There is a risk that replication failures might be overestimated by using original *p*-values due to the wide sample-to-sample variability and influence of sample size [47, 48]. Consequently, the “vote counting” method, whereby replication is simply assessed on whether or not it has reached significance compared to the original study [42], will not be solely used to assess replication outcomes as it does not completely represent evidence of replicability [49]. Accordingly, the replication effect size and 95% confidence limits will also be plotted against the original effect size as discussed previously.

Lastly, it seems necessary to justify the non-use of a Bayesian decision-making framework for selecting replication studies due to the support for its use in the literature. Bayesian analysis can be useful as it evaluates the probability that a claim is robust based on the combination of the new study evidence with the plausible prior distribution of the data [50]. However, the plausible prior distribution of the data depends on the prior evidence, therefore, the use of a default prior may not be possible due to high publication bias [24]. A Bayesian decision-making framework may possibly be of higher value for a single study replication as a default prior could be used, or a literature review could be conducted on each topic or claim to inform the prior. However, assuming that sufficient prior information is available for every replication study in this protocol would be difficult to justify. A Bayesian decision-making framework is simply not feasible for a large-scale project with many sub-disciplines; it also requires additional expertise which may not be available to replication teams.

A limitation of this study selection protocol is the focus on quartile 1 journals for replication. Many researchers are driven by the “publish or perish” attitude and tend to embrace research topics that will produce strong or novel effects for publication in higher ranked journals [51]. The focus on the “prestigious” journals of quartile 1 rankings in this protocol may lead to replications of potentially overestimated effect sizes [20]. We acknowledge that the eligibility criteria in this protocol is of sufficient detail to allow for a replication attempt with methods as close as possible to the original study methods, i.e. a close replication [9]. It thereby excludes studies lacking sufficient detail. As a result, this replication project should not be considered a final evaluation of the replicability of sports and exercise science research but rather an initial estimate of the upper limit of replicability of the field.

The collection of studies accumulated in the study selection pool will not, of course, be completely representative of the entirety of sports and exercise science research. This is aptly described by Stroebe as an impossible task because a true representative sample would include both published and non-published research [21]. One must then focus on the replicability of published research even with the high potential for publication bias. Even still, the pool of studies will be exceedingly large if inclusive of all sports and exercise science research; hence, the starting point of modern-day research.

## Conclusion

The key purpose of this paper was to transparently describe and justify a selection protocol for replication studies in sports and exercise science. This selection protocol will be used for a planned large-scale replication effort in sports and exercise science and could be adopted by other replication teams.

## Supplementary Information

Below is the link to the electronic supplementary material.

Supplementary file1 (DOCX 20 KB)
