# Supplementary material for: Proposal of a Selection Protocol for Replication of Studies in Sports and Exercise Science
Source: Sports Med. 2022 Sep 6;53(1):281–91. doi: 10.1007/s40279-022-01749-1 (PMC9807474; doi:10.1007/s40279-022-01749-1)
Supplement: Supplementary file 1 — Supplementary file1 (DOCX 20 KB) [file 40279_2022_1749_MOESM1_ESM.docx]

# Supplementary Information

# Reference List Quartile Ranking Assessment

The purpose of this task was to identify which quartile ranking articles in published reference list were most cited from. This will then inform the protocol for the selection of studies for replication.

## Methods

The steps for this task were completed as follows:

1. The journal rankings list was selected on [www.scimagojr.com](http://www.scimagojr.com)
2. It was filtered for “sports science” and the year “2019” (as “2020” rankings were not available as of 30^th^ October 2020)
3. The top 5 journals and lowest ranked journal of each quartile were selected for the assessment of their reference lists
4. If the journal language was not English or if the reference lists were unavailable, this journal was excluded, and the next journal was selected
5. The specific journal website (e.g. <https://bjsm.bmj.com>) was accessed and the volume for 2019 was selected
6. In reverse chronological order, the issue was selected (i.e. the last issue of the year - December);
   1. An article within that issue was also selected in reverse chronological order (i.e. the last dated article of that issue)
   2. If there were no timestamps available, the first listed article was selected
   3. If the article reference list was unavailable to access, the open access article of that issue was then selected
7. The reference list of that article was assessed;
   1. The journal name from each reference on the list was searched on [www.scimagojr.com](http://www.scimagojr.com) for the quartile ranking allocated to that journal in 2019
   2. The best quartile ranking of the journal for that year was entered into Microsoft Excel (version 16.43) for that reference (e.g. q1(quartile 1 for reference 1)
   3. If there was no journal ranking available on [www.scimagojr.com](http://www.scimagojr.com) for that article or if it has no ranking as it is a book, “No SJR” was entered into the spreadsheet to indicate “No Scimago journal ranking”
8. On completion of the reference list in the article of that issue, the previous issue was selected and step 6 and 7 were repeated for the reference list of another article (e.g. November issue)
9. When the reference lists for each journal were completed, the total distribution of quartile rankings for that reference list were totalled using the count function in Excel e.g. =COUNTIF(A1:K1, “q1”)
10. The total count was converted to quartile percentages based on the total references for that article and for that journal
11. The total references for each journal and the percentage quartile breakdown of their reference lists are shown in Table 1

Only original articles were included in this task therefore reviews, editorials and consensus statements were excluded as these will also be excluded from the study selection pool for replication.

## Results

A total of 50 journal article reference lists were assessed in each quartile (total N = 200). Across all 4 quartiles, authors most cited other journals that were ranked in quartile 1 according to [www.scimagojr.com](http://www.scimagojr.com) (see Table 1).

| Table 1 Mean citation pattern of reference lists in published articles by [www.scimagojr.com](http://www.scimagojr.com) quartile ranking | | | | | |
| --- | --- | --- | --- | --- | --- |
|  | **Articles referenced from quartile 1** | **Articles referenced from quartile 2** | **Articles referenced from quartile 3** | **Articles referenced from quartile 4** | **Articles referenced which had no ranking** |
| Articles published in quartile 1 | 77.93 % | 8.39 % | 2.92 % | 0.36 % | 10.40 % |
| Articles published in quartile 2 | 69.71 % | 8.80 % | 3.36 % | 0.48 % | 17.66 % |
| Articles published in quartile 3 | 58.08 % | 19.63 % | 5.33 % | 0.65 % | 16.31 % |
| Articles published in quartile 4 | 53.17 % | 12.71 % | 9.49 % | 2.92 % | 21.71 % |
| For all articles  (N = 200) | 64.72 % | 12.38 % | 5.27 % | 1.10 % | 16.52 % |

For articles published in journals ranked in quartile 1 according to [www.scimagojr.com](http://www.scimagojr.com), 77.93% of the reference lists cited quartile 1 journals. For articles published in journals ranked in quartile 2, 69.71% of the reference lists cited quartile 1 journals. For articles published in journals ranked in quartile 3, 58.08% of the reference lists cited quartile 1 journals. For articles published in journals ranked in quartile 4, 53.17% of the reference lists cited quartile 1 journals.

## Conclusion

The aim of this task was to identify the most cited quartile ranking for journals used in reference lists. The results of this task show that journal articles which are published in quartile 1 journals, according to [www.scimagojr.com](http://www.scimagojr.com), are the most frequently cited across articles published in all 4 quartiles. This information will be employed in the study selection protocol as one of the selection criteria.
